# Effects of remimazolam on hemodynamics in children with congenital heart disease undergoing cardiac catheterization

**DOI:** 10.3389/fphar.2025.1714645

**Published:** 2025-11-24

**Authors:** Hongyun Li, Zhaomeng Song, Xunwei Jiang, Wei Liu, Yan Jiang, Rong Wei

**Affiliations:** 1 Department of Anesthesiology, Shanghai Children’s Hospital, School of Medicine, Shanghai Jiao Tong University, Shanghai, China; 2 Department of Cardiology, Shanghai Children’s Hospital, School of Medicine, Shanghai Jiao Tong University, Shanghai, China

**Keywords:** remimazolam, child, hemodynamics, congenital heart defects, cardiac catheterization

## Abstract

**Introduction:**

Maintaining hemodynamic stability during anesthesia is crucial for patients with congenital heart disease. Remimazolam, a novel benzodiazepine, offers advantages, such as rapid onset, quick recovery, stable hemodynamics, and mild respiratory depression. We aimed to assess the effects of a single intravenous dose of remimazolam on hemodynamics in children with congenital heart disease.

**Methods:**

This self-controlled before-and-after study enrolled 40 children undergoing elective cardiac catheterization and transcatheter interventional closure at Shanghai Children’s Hospital between June and September 2024. No special preoperative medications were administered. After entering the operating room, noninvasive hemodynamic parameters, such as heart rate (HR), mean arterial pressure (MAP), oxygen saturation (SPO_2_), cardiac output (CO), and cardiac index (CI) were monitored. During the cardiac catheterization procedure, invasive hemodynamic parameters—including superior vena cava pressure (SVCP), right atrial pressure (RAP), right ventricular pressure (RVP), and pulmonary artery pressure (PAP)—were measured according to surgical requirements. Subsequently, remimazolam (0.3 mg/kg) was administered intravenously, and the same parameters were remeasured 3 min later. The impact of remimazolam on hemodynamics in children with CHD was evaluated by comparing the changes in these indicators before and after drug administration.

**Results:**

Five patients were excluded due to incomplete data, leaving 35 for analysis (sex, 11 male, 24 female; median age, 6.67 [interquartile range: 4–11.5 years]). Following intravenous administration of remimazolam, all non-invasive hemodynamic parameters remained stable, showing no statistically significant differences before versus after medication: HR [(104.31 ± 20.27) vs. (104.91 ± 19.76) bpm, P = 0.485], MAP [61 (58, 65) vs. 61 (57, 66) mmHg, P = 0.313], CO [3.2 (2.34, 3.5) vs. 3 (2.41, 3.7) L/min, P = 0.133], and CI [3.3 (3, 3.6) vs. 3.3 (3, 3.71) L/min/m^2^, P = 0.292]. Similarly, no statistically significant differences were observed in right heart system pressures before and after administration: mRAP [8 (6, 10) vs. 8.5 (6.75, 10.25) mmHg, P = 0.064] and mPAP [15.5 (14, 19) vs. 16 (14, 19.5) mmHg, P = 0.517]. No adverse reactions such as bradycardia, hypotension, or hypertension were observed after intravenous injection of remimazolam.

**Conclusion:**

During sevoflurane-maintained anesthesia, co-administration of remimazolam provides good hemodynamic stability for children with left-to-right shunt congenital heart disease undergoing cardiac catheterization.

## 1 Introduction

Children with congenital heart disease (CHD) are at increased risk of hemodynamic instability during general anesthesia. Anesthesia induction in these patients prioritizes maintaining hemodynamic stability, controlling myocardial oxygen consumption, and minimizing procedural stress (Lee et al., 2024). Currently, propofol and etomidate are commonly used for anesthesia induction in pediatric patients with CHD. While propofol is a rapid-acting sedative that allows quick recovery, it is associated with a high incidence of hypotension, bradycardia, and injection pain (Fleck et al., 2014; Vullo et al., 2024). Etomidate exerts minimal effects on hemodynamics and does not cause dose-dependent hypotension (Spanos and Tye, 1978); however, it carries the risk of adrenal suppression and myoclonus (Komatsu et al., 2013; Hu et al., 2023).

Remimazolam exerts its effect by agonizing the GABA-A receptors, and has an onset of action of 1–3 min, (Hu et al., 2022), offers several advantages over propofol and etomidate, including rapid onset, fast metabolism, hemodynamic stability, minimal circulatory fluctuation, mild respiratory depression, and reversibility (Gao et al., 2023). Compared with propofol, remimazolam has a lower risk of cardiovascular depression, respiratory depression, and injection pain (Kilpatrick, 2021; Kim, 2022). Similarly, compared to etomidate, it is associated with less injection pain and reduced myoclonus (Hu et al., 2023).

Remimazolam has been used safely as a sedative and anesthetic in patients with cardiac disease (Ripoll et al., 2025), including those with aortic valve stenosis (Furuta et al., 2021), those undergoing valve replacement (Liu et al., 2021), or those undergoing radiofrequency ablation for atrial fibrillation (Nam et al., 2023). Moreover, it has been employed during cardiovascular procedures (Aoki et al., 2022) and various diagnostic and interventional procedures (Gillis et al., 2024). However, use of remimazolam in pediatric CHD remains unsupported by large randomized controlled trials. Therefore, in this prospective observational study, we aimed to evaluate these effects by comparing hemodynamic changes before and after a single administration of remimazolam.

## 2 Methods

### 2.1 Study design and patients

**FIGURE 1:**
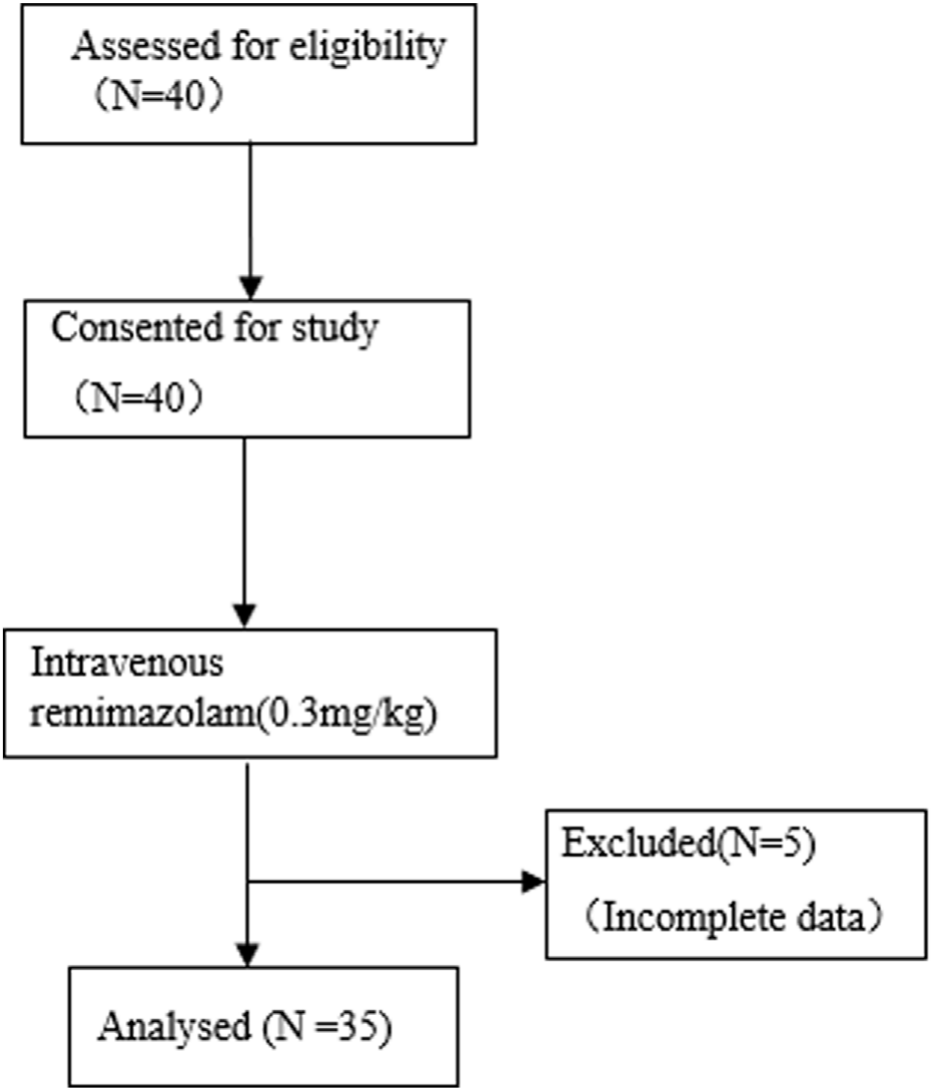
Flow chart of patient allocation.

This study evaluated the effects of a single intravenous injection of remimazolam on the hemodynamic parameter changes in children with CHD. The study was a single-center, self-controlled before-and-after study, approved by the Ethics Committee of Shanghai Children’s Hospital (2024R059-F01) and registered at the Chinese Clinical Trial Registry (chictr.org.cn; Registration No.: ChiCTR2400084993, Registration Date: 29 May 2024). All procedures were carried out in accordance with the Declaration of Helsinki. Informed consent was obtained from the parents of 40 children with CHD who were scheduled to undergo cardiac catheterization and transcatheter closure. However, due to incomplete data from five patients, only 35 patients were included in the final analysis. The study flow is illustrated in Figure 1.

#### 2.1.1 Inclusion and exclusion criteria

The inclusion criteria were as follows: age <18 years with American Society of Anesthesiologists (ASA) classification II or III and no premedication. Patients meeting these criteria were scheduled for elective cardiac catheterization and transcatheter closure under general anesthesia.

Patients were excluded if they had a known allergy to benzodiazepines, were taking sedatives or anticonvulsant medications, had a history of liver or kidney dysfunction or other systemic complications prior to the procedure, individuals with incomplete data collection.

#### 2.1.2 Anesthesia induction and maintenance

All patients did not receive any special medication before the operation. After entering the operating room, routine intraoperative monitoring was performed using a multi-parameter electrocardiogram monitor (Model AM, Serial No. 6955816; DIVA Laboratories Ltd., Taipei, Taiwan) to assess electrocardiogram, non-invasive blood pressure (BP), and oxygen saturation (SpO_2_). Continuous monitoring of cardiac output (CO) and cardiac index (CI) was conducted using a non-invasive CO monitor (Model ICON(C3); Shanghai Meta Care Medical Device Co. Ltd., Shanghai, China).

Anesthesia was induced intravenously with glycopyrrolate 4 μg/kg (maximum 100 μg), propofol 2–3 mg/kg, rocuronium 0.6 mg/kg, and sufentanil 0.2 μg/kg. Following induction, a laryngeal mask airway was inserted to facilitate mechanical ventilation. Anesthesia was maintained with pressure-controlled ventilation, targeting an end-tidal CO_2_ (EtCO_2_) of 35–45 mmHg. Sevoflurane (2%–3%) was continuously inhaled to maintain a minimum alveolar concentration (MAC) of 1–1.3.

### 2.2 Data measurement and classifications

After general anesthesia was established and mechanical ventilation was initiated, continuous monitoring of heart rate (HR), blood pressure (BP), SpO_2_, cardiac output (CO), and cardiac index (CI) was performed. During cardiac catheterization, anesthesiologists recorded invasive hemodynamic parameters including superior vena cava pressure (SVCP), right atrial pressure (RAP), right ventricular pressure (RVP), and pulmonary artery pressure (PAP). Remimazolam tosilate for injection (Jiangsu Hengrui Pharmaceuticals Co., Ltd., Beijing, China; 25 mg/vial; Batch No: 231123AK; National Drug Approval No: H20217078) was administered intravenously at a dose of 0.3 mg/kg. Three minutes after administration, the above parameters were re-measured.

The primary outcome measures included changes in non-invasive hemodynamic parameters (HR, MAP, CO, CI) and changes in invasive hemodynamic parameters (mean of SVCP, RAP, RVP, PAP) before and after drug administration. Secondary outcomes included mean PAP (mPAP) and adverse cardiovascular events, such as bradycardia, hypotension, and hypertension. Bradycardia was defined as HR ≤50 bpm. Hypotension was defined as a ≥30% reduction in mean arterial pressure (MAP) from baseline. Hypertension was defined as a ≥30% increase in MAP from baseline.

### 2.3 Statistical analysis

Data were analyzed using the SPSS software (version 26.0; IBM Corp., Armonk, NY, United States). Normality was assessed using the Shapiro–Wilk test. Normally distributed data are presented as means ± standard deviations; non-normally distributed data as medians (interquartile ranges) [M (IQR)]. Paired t-tests or Wilcoxon signed-rank tests were used to compare pre- and post-treatment values. A p-value <0.05 was considered statistically significant.

The sample size for this study was determined with reference to the statistical methodology described by Sarkar et al. (2005) in their study on the hemodynamic effects of etomidate. Based on the mean and standard deviation of the differences in hemodynamic parameters before and after drug administration, and using a paired-design t-test with α = 0.05 (two-sided) and β = 0.05 (i.e., a statistical power of 95%), calculation with PASS 15 software indicated that a sample size of 30 patients would achieve the desired power. This study ultimately enrolled 35 patients, which exceeds the minimum sample size requirement and is sufficient to meet the needs of the statistical analysis.

## 3 Results

### 3.1 Characteristics and demographic data of the patients

A total of 40 pediatric patients were recruited according to the inclusion and exclusion criteria. Due to incomplete data from five patients, 35 were included in the final analysis: 11 male and 24 female individuals, with a median age of 6.67 (IQR: 4–11.5) years, median weight of 20.5 kg (IQR: 15–35), and median body mass index (BMI) of 15.28 kg/m^2^ (IQR: 13.97–16.96). Among them, 20 had atrial septal defects (ASD), eight had ventricular septal defects (VSD), six had patent ductus arteriosus (PDA), and one (female, 2.25 years old, 15 kg, BMI 15.94) had both ASD and VSD. Baseline characteristics are presented in Table 1.

**TABLE 1:** Baseline clinical characteristics of the study participant.

Variable		All (N = 35)	VSD (N = 8)	PDA (N = 6)	ASD (N = 20)
Sex	Female	24 (68.57%)	3 (37.5%)	4 (66.67%)	16 (80%)
	Male	11 (31.43%)	5 (62.5%)	2 (33.33%)	4 (20%)
Age (y)		6.67 (4, 11.5)	4.46 (3.00, 6.71)	5.92 (3.27, 9.12)	7.38 (5.40,13.69)
Weight (kg)		20.5 (15, 35)	17 (15, 20.38)	19.25 (15, 24.13)	27.75 (16.13,52.38)
BMI (kg/m^2^)		15.28 (13.97, 16.96)	15.12 (14.38, 16.02)	14.26 (13.36, 16.21)	15.88 (13.93,20.45)

Abbreviations: ASD, atrial septal defect; VSD, ventricular septal defect; PDA, patent ductus arteriosus.

### 3.2 Primary outcome measures

#### 3.2.1 Non-invasive hemodynamic parameter changes

**FIGURE 2:**
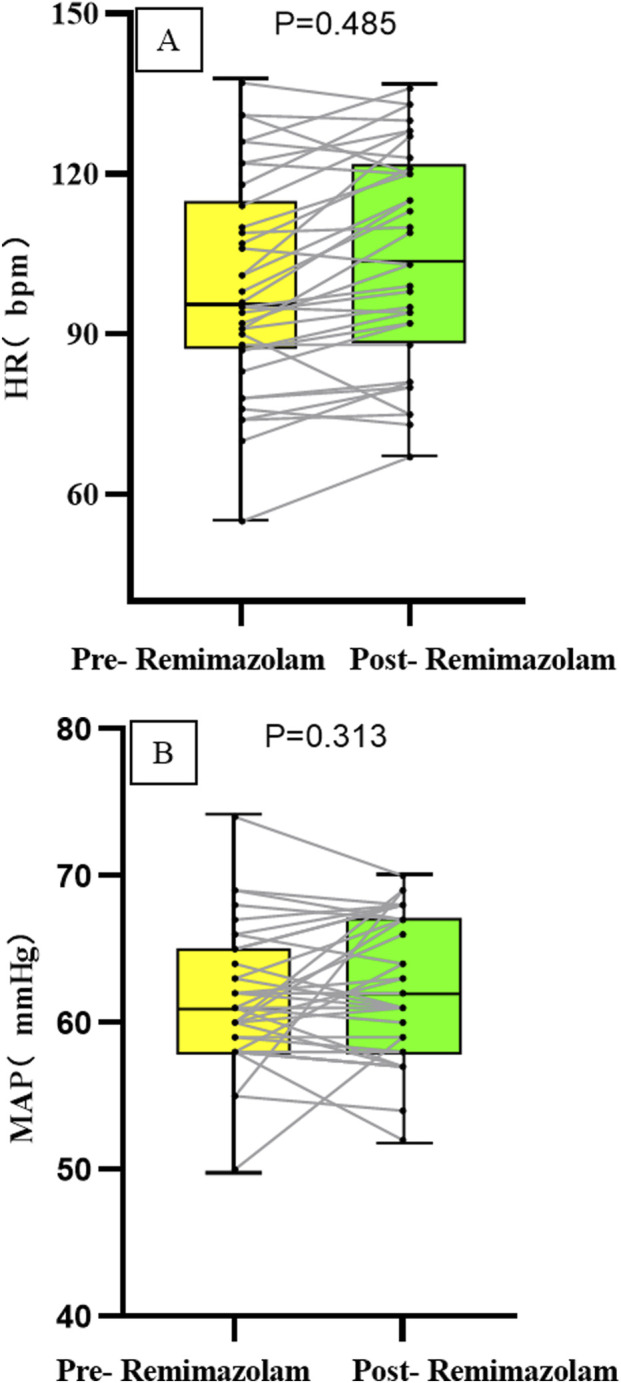
Changes in the absolute values of HR **(A)** and MAP **(B)** in 35 children.

The heart rate (HR) after intravenous administration of remimazolam was (104.91 ± 19.76) pbm, compared to [(104.31 ± 20.27) pbm] before administration, with no statistically significant difference (P = 0.485). The mean arterial pressure (MAP) after intravenous remimazolam administration was 61 (57, 66) mmHg, compared to [61 (58, 65) mmHg] before administration, showing no statistically significant difference (P = 0.313). Moreover, the fluctuations in both HR and MAP before and after drug administration were less than 20%; the absolute changes are presented in Figure 2.

**FIGURE 3:**
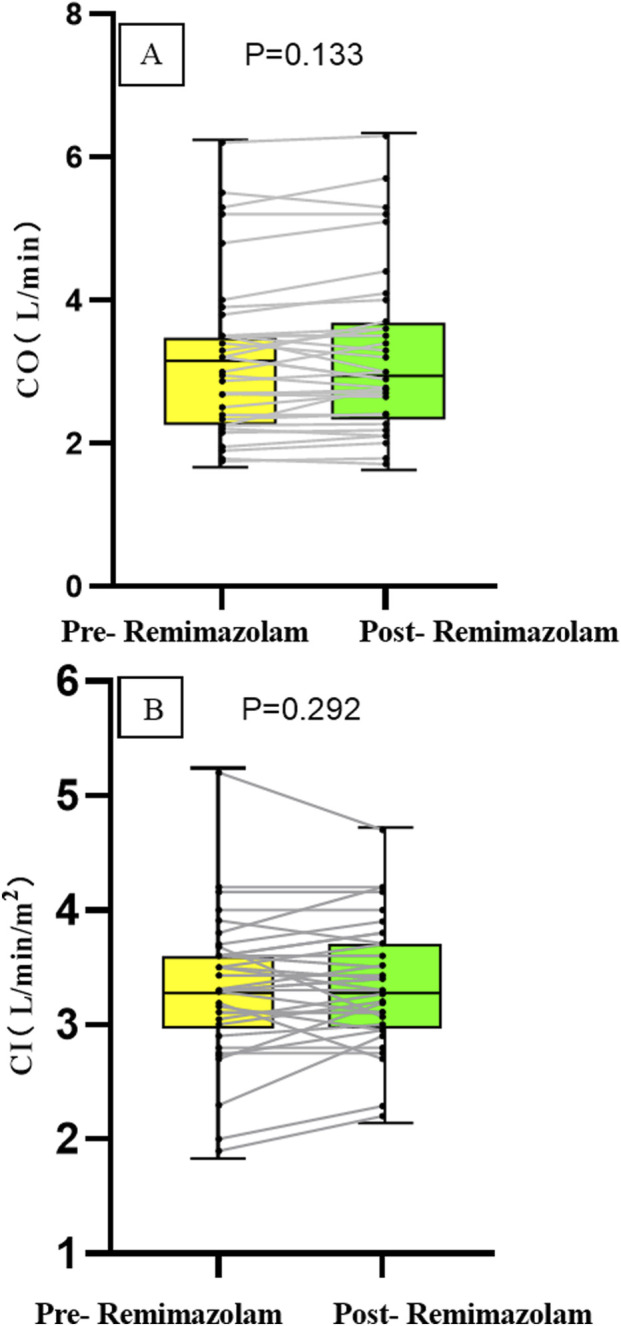
Changes in the absolute values of CO **(A)** and CI **(B)** in 35 children.

The cardiac output (CO) after intravenous administration of remimazolam was 3.0 (2.41, 3.7) L/min, compared to [3.2 (2.34, 3.5) L/min] before administration, with no statistically significant difference (P = 0.133). The cardiac index (CI) after intravenous remimazolam administration was 3.3 (3.0, 3.71) L/min/m^2^, compared to [3.3 (3.0, 3.6) L/min/m^2^] before administration, showing no statistically significant difference (P = 0.292). After intravenous administration of remimazolam, one patient with VSD exhibited a 20% increase in CO and CI compared to pre-administration values, while the fluctuations in CO and CI remained within 20% in all other patients, the absolute changes are presented in Figure 3.

#### 3.2.2 Invasive hemodynamic parameter changes

The mean superior vena cava pressure (mSVCP) after intravenous remimazolam administration was 9 (6, 10.5) mmHg, compared to [8 (6, 11) mmHg] before administration, with no statistically significant difference (P = 0.173). The mean right atrial pressure (mRAP) was 8.5 (6.75, 10.25) mmHg after administration, compared to [8 (6, 10) mmHg] before, showing no statistically significant difference (P = 0.064). The mean right ventricular pressure (mRVP) was 9 (16, 11.25) mmHg after administration, versus [9 (6, 12) mmHg] before, with no statistically significant difference (P = 0.877). The mean pulmonary artery pressure (mPAP) was 16 (14, 19.5) mmHg after administration, compared to [15.5 (14, 19) mmHg] before, and the difference was not statistically significant (P = 0.517) (Table 2).

**TABLE 2:** Comparison of hemodynamic variables between pre- and post-administration of remimazolam for all 35 patients.

Variable	Pre-remimazolam	Post-remimazolam	95% CI	P value
HR (bpm)	104.31 ± 20.27	104.91 ± 19.76	−2.326, 1.126	0.485
MAP (mmHg)	61 (58, 65)	61 (57, 66)	0.267, 0.284	0.313
CO(L/min)	3.2 (2.34, 3.5)	3 (2.41, 3.7)	0.127, 0.140	0.133
CI(L/min/m^2^)	3.3 (3, 3.6)	3.3 (3, 3.71)	0.293, 0.311	0.292
mSVCP (mmHg)	8 (6, 11)	9 (6, 10.5)	0.168, 0.183	0.173
mRAP (mmHg)	8 (6, 10)	8.5 (6.75, 10.25)	0.06, 0.07	0.064
mRVP (mmHg)	9 (6, 12)	9 (16, 11.25)	0.875, 0.888	0.877
mPAP (mmHg)	15.5 (14, 19)	16 (14, 19.5)	0.506, 0.526	0.517

*P<0.05; 95% CI, 95% Confidence Interval; HR, heart rate; MAP, Mean arterial, CO, cardiac output; CI, cardiac index; mSVCP, mean superior vena cava pressure; mRAP, mean right atrial pressure; mRVP mean right ventricular pressure; mPAP, mean pulmonary artery pressure.

### 3.3 Secondary outcomes

**FIGURE 4:**
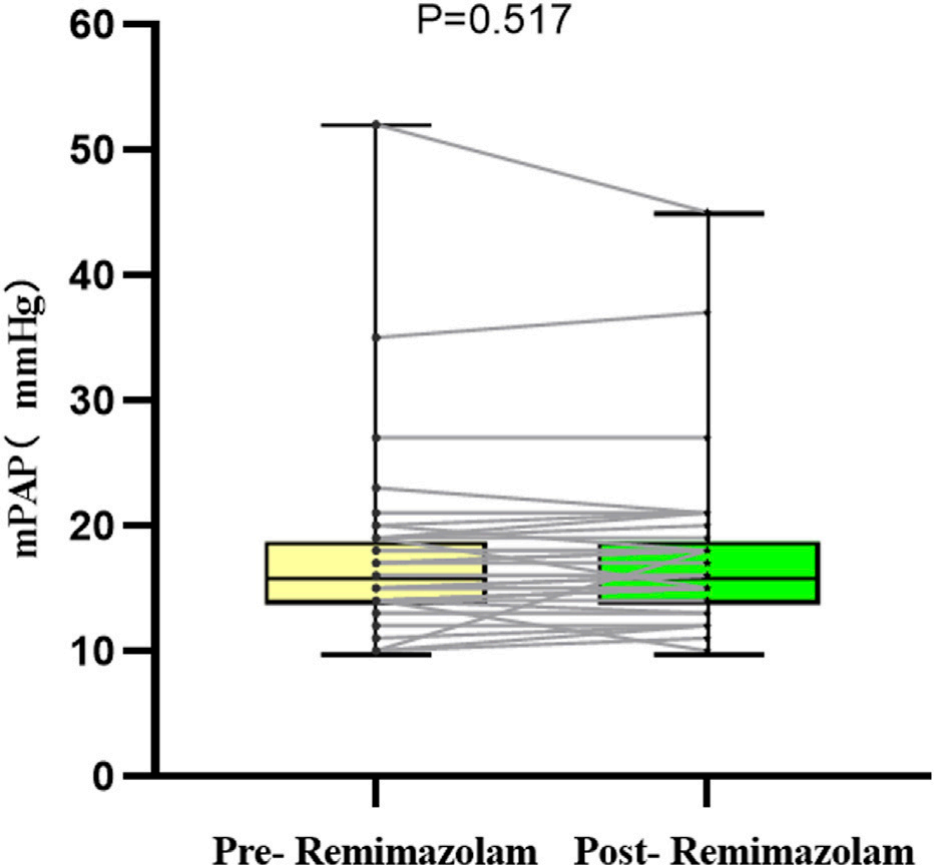
Changes in the absolute value of mPAP in 35 children. mPAP, mean pulmonary artery pressure.

Pulmonary hypertension was defined as mPAP >20 mmHg (Humbert et al., 2023), Five patients met this criterion prior to remimazolam administration, No significant changes in mPAP were observed pre- and post-treatment in these five patients (p = 0.750, Table 3). Similarly, in the overall cohort of 35 patients, there was no significant difference in mPAP following a single intravenous injection of remimazolam (p = 0.517, Figure 4). Importantly, no adverse events, such as bradycardia, hypotension, or hypertension, were reported.

**TABLE 3:** Changes of mPAP in patients with pulmonary hypertension pre- and post-administration of remimazolam for 5 patients.

Diagnosis	mPAP (mmHg) pre-remimazolam	mPAP (mmHg) post-remimazolam	95% CI	P value
PDA	35	37	0.741, 0.758	0.414
VSD	52	45		
PDA	23	21		
ASD	21	21		
ASD	27	27		

## 4 Discussion

This study was a self-controlled before-and-after design,it analyzed the effects of a single intravenous injection of remimazolam on hemodynamic parameters through non-invasive and invasive methods in children with congenital heart disease (CHD) during cardiac catheterization. Drug dosage is a significant risk factor for drug-related adverse effects in children with CHD (Toni et al., 2025). The trial dosage of remimazolam in this study was set at 0.3 mg/kg. This dosage was determined based on a comprehensive consideration of its safety profile, clinical experience, and pharmacokinetic characteristics: as a GABA-A receptor agonist, remimazolam has an onset of action of 1–3 min (Hu et al., 2022). Chae et al. (2022) recommended the optimal bolus dosing based on the 95% effective dose: 0.25–0.33 mg/kg. Therefore, hemodynamic assessment was performed 3 min after drug administration. The results demonstrate that remimazolam exhibited favorable hemodynamic stability in children with left-to-right shunt CHD, such as atrial septal defect (ASD), ventricular septal defect (VSD), orpatent ductus arteriosus (PDA). The results demonstrated that a 0.3 mg/kg dose of remimazolam did not cause any statistically significant changes in both non-invasive (HR, MAP, CO, CI) and invasive (mSVCP, mRAP, mRVP, mPAP) hemodynamic parameters, all remained stable and unchanged. Furthermore, no drug-related adverse events, including hypotension, hypertension, or bradycardia observed.

These findings are consistent with previous studies in adult cardiac surgery populations. For instance, Liu et al. (2021) reported that a single bolus of 0.3 mg/kg remimazolam caused significantly less hemodynamic fluctuation compared to propofol in cardiac surgery patients. In our study, continuous monitoring of HR and MAP via a multi-parameter monitor showed that the magnitude of change for both parameters before and after drug administration was less than 20%, with no statistically significant difference observed, further supporting the favorable hemodynamic stability of remimazolam in children with CHD undergoing cardiac catheterization.

Studies have indicated that chronic comorbidities are among the most common risk factors for drug-related adverse effects in children (Toni et al., 2024). Due to immature liver and kidney function in children, drug metabolism and excretion may be altered, thereby increasing their susceptibility to drug-related side effects (Fernandez et al., 2011). Children with CHD may experience altered hepatic and renal function secondary to cardiac dysfunction. However, remimazolam is primarily metabolized by tissue esterases via hydrolysis and is independent of hepatic or renal function. Therefore, its impact on the pathophysiology of CHD is likely limited.

However, it is noteworthy that the changes in HR and MAP observed in our study differ from the findings reported by Kimoto et al. (2023) in children under general anesthesia. The latter study noted that the majority of their pediatric subjects experienced fluctuations in blood pressure and heart rate exceeding 20%. The potential reasons for this discrepancy may include the following two aspects: First, the administration of remimazolam in our study was based on a well-established background anesthetic regimen (maintained with sevoflurane), which effectively mitigated sympathetic excitation caused by factors such as anxiety and crying. Second, our study employed a single intravenous bolus, whereas Kimoto et al. utilized a continuous infusion. Given that remimazolam exhibits first-order pharmacokinetics and its sedative effect is dose-dependent (Doi et al., 2020), a single bolus administration may be more conducive to maintaining hemodynamic stability. Furthermore, the concomitant use of opioids (such as a rapid fentanyl bolus) in the Kimoto study could itself have contributed to a decrease in blood pressure, thereby confounding the isolated hemodynamic impact of remimazolam.

In the surgical management of children with CHD, beyond providing sedation and analgesia, a key consideration in anesthetic drug selection is to minimize impacts on hemodynamic and electrophysiological functions (Lam et al., 2014). In our study, a non-invasive CO monitor was used for continuous CO and CI assessment, offering a minimally invasive approach. The results indicated no statistically significant differences in CO and CI before and after drug administration. From a pharmacological perspective, the relatively stable hemodynamic profile of remimazolam may be attributed to its limited effects on myocardial contractility and systemic vascular resistance. Tang et al. (2021) demonstrated that remimazolam likely contributes to circulatory stability by modulating surgical stress responses and mildly enhancing myocardial contractility. Similarly, Qiu et al. (2022) suggested that its minimal influence on blood pressure may be related to its negligible effects on cardiac output and systemic vascular resistance. This mechanism is particularly important in children with CHD, who require delicate hemodynamic management.

A significant strength of this study lies in the simultaneous use of both non-invasive and invasive monitoring methods. The invasive monitoring data were entirely derived from routine clinical procedures, providing direct evidence for evaluating the effects of remimazolam on right heart pressure parameters without imposing additional trauma on the children. The results demonstrated that a single intravenous injection of remimazolam did not significantly affect mean SVCP, mean RAP, mean RVP, or mean PAP in children with CHD, indicating its minimal impact on right heart function and pulmonary vascular resistance. Yoshikawa et al. (2024) found that benzodiazepines such as remimazolam typically bind and agonize the g subunit of GABA-A, cardiac-specific GABAA receptors have a lower expression of g subunits and, therefore, exhibit a weaker binding of benzodiazepines. This finding may inpart help to explain why benzodiazepines have a milder effect on cardiac contractility.

Pulmonary hypertension (PH) is a crucial parameter for assessing the status of the pulmonary vascular bed, surgical indications, and postoperative recovery in children with CHD. It is noteworthy that this study included five children with comorbid PH, and the results demonstrated that remimazolam did not exert a significant impact on their mPAP. Furthermore, no statistically significant difference in mPAP was observed before and after drug administration even in CHD children with normal preoperative pulmonary artery pressure. This finding holds considerable clinical significance, may suggesting that remimazolam may be suitable for anesthetic management in CHD children complicated with PH. However, further validation in larger cohorts is required to confirm this observation.

In summary, the co-administration of remimazolam during sevoflurane-based maintenance anesthesia can provide favorable hemodynamic stability for children with left-to-right shunt congenital heart disease undergoing cardiac catheterization. It is important to note that these findings are applicable specifically to this patient subgroup and anesthetic regimen (sevoflurane with remimazolam), and should not be generalized to all children with CHD in the absence of further randomized studies.

### 4.1 Limitations

This study has several limitations. First, it employed a self-controlled before-and-after design. While this approach effectively reduces inter-subject variability, it lacks a parallel control group. Consequently, we cannot entirely attribute the observed hemodynamic changes solely to the effect of remimazolam. Second, time-varying confounding factors, such as surgical stimulation, depth of anesthesia, and the cumulative effects of other anesthetic agents, may have collectively influenced the hemodynamic parameters. Therefore, the hemodynamic stability associated with remimazolam observed in this study should be interpreted as the combined effect of multiple factors. Future validation through more rigorously designed and controlled randomized clinical trials is warranted.

## Data Availability

The original contributions presented in the study are included in the article/supplementary material, further inquiries can be directed to the corresponding authors.
